# Scheduling, Income, and School Closures: Unlocking the Key Drivers of Home Care Personal Support Unplanned Absences Through Time-to-Event Regression Analysis

**DOI:** 10.1177/07334648251316973

**Published:** 2025-02-06

**Authors:** Katherine A. P. Zagrodney, Rachael Jaffe, Sandra M. McKay, Kashmeena Mangal, Travis A. Van Belle, Kathryn A. Nichol, Emily C. King

**Affiliations:** 1 VHA Home HealthCare, Toronto, ON, Canada; 2 Canadian Health Workforce Network (CHWN), University of Ottawa, Ottawa, ON, Canada; 3 Institute of Health Policy Management and Evaluation (IHPME), 7938 University of Toronto, Toronto, ON, Canada; 4 Ted Rogers School of Management, Toronto Metropolitan University, Toronto, ON, Canada; 5 Faculty of Arts and Science, 7938 University of Toronto, Toronto, ON, Canada; 6 Dalla Lana School of Public Health (DLSPH), 7938 University of Toronto, Toronto, ON, Canada; 7 School of Public Health Sciences, University of Waterloo, Waterloo, ON, Canada

**Keywords:** caregiving, health services, home and community-based care and services, home care, staff shortages, health workforce, time-to-event regression models, healthcare labor supply, COVID-19

## Abstract

Healthcare workers’ unplanned workplace absences are a global challenge with consequences for care recipients, employers, and healthcare systems. With rising home care demand, understanding drivers of home care personal support worker (PSW) absences can inform management of labor supply through government and employer policies. In this paper, we examined predictors of PSWs’ unplanned absences within a large, administrative, longitudinal dataset (2019–2021) from a home care organization in Ontario, stratified by time (pre-, early-, and mid-pandemic). After an initial spike, unplanned absence rates were generally lower during the pandemic. Cox-proportional hazard regression models for unplanned absences highlighted how increasing income and reducing travel distance between visits can be expected to decrease the hazard of unplanned absences. School closures significantly correlated with unplanned absences, highlighting disruptions within the broader care economy. As demand for home care accelerates, reducing unplanned absences will improve care consistency for those relying on PSWs to remain safe at home.

**What this paper adds**

• This paper provides insight into factors of influence on home care personal support workers (PSWs) unplanned workplace absences, a previously under-researched topic of high relevancy to health workforce planning given the increased demands on home care due to an aging population.
• Using a large longitudinal dataset which contained detailed workplace variables often not captured in prior literature, we found that key contributing factors for PSW unplanned absences were lower income and longer travel distance between care visits.
• Findings revealed how this highly gendered workforce, whose members are more likely to have additional unpaid family caregiving responsibilities, responded during periods of school closures.

**Application of study findings**

• As demand for home care continues to grow, improving stability of home care access and PSW supply through reducing unplanned absences will be increasingly beneficial for PSW themselves and for those relying on PSW care to remain safe at home.
• Based on study findings, policies aimed at increasing income and reducing travel distance between clients are expected to improve home care PSW labor supply via reductions to unplanned absences.

## Background

Unplanned absences amongst healthcare workers is a global challenge, with negative consequences for healthcare workers (Brady et al., 2023a), those seeking care (CIHI, 2023), employers (Kocakulach et al., 2016), and society (Gianino et al., 2019). Healthcare workers tend to have higher levels of unplanned work absences than other occupations, a trend which intensified during the COVID-19 pandemic (e.g., unplanned absences due to illness/disability increased by 23% from 2019 to 2020 for healthcare workers vs. 9% for other occupations (Blackwell, 2023).

Rates of unplanned absences vary by healthcare occupational groups, with personal support workers (PSWs) (also known as direct care workers, health care aides, and other titles dependent on jurisdiction) generally experiencing higher rates of unplanned absences compared to other healthcare workers, prior to and during the COVID-19 pandemic (Blackwell, 2023; Gorman et al., 2010). The relatively high rates of unplanned absences for PSWs can have a particularly negative impact on labor supply in the home care sector, where PSWs are heavily relied upon to provide the majority (70%–80%) of paid direct care services (Sinn et al., 2022), including helping older adults with activities of daily living at home (e.g., eating and bathing). Home care PSW labor supply challenges are substantial and chronic and were exacerbated by the pandemic (Global Coalitation on Aging & Home Instead, 2021; King et al., 2023; Ontario Community Support Association (OCSA), 2022). At the same time that the workforce grapples with existing shortages, projections indicate that the demand for home care services will continue to grow (Deloitte, 2021; Global Coalitation on Aging & Home Instead, 2021).

As in other countries, home care PSW labor shortages were a key challenge during the pandemic in Canada (Nizzer et al., 2023). Throughout the pandemic, Canadian provincial governments looked to directly address PSW labor supply by enacting PSW-specific wage enhancement policies (Government of British Columbia, 2020; Government of Saskatchewan, 2020; Government of Ontario, 2020). At the same time, other pandemic policies enacted to limit the spread of COVID-19 may have had unintended consequences for PSW labor supply. For instance, school and daycare closures increased childcare responsibilities for parental figures and limited their ability to work outside of the home (Gallagher-Mackay et al., 2021). School closures also corresponded with a pause in access to other services, such as seniors’ centers and older adult community day services, which impacted family caregivers of older adults (Wister & Kadowaki, 2021). Evidence-based health workforce planning requires understanding the factors that contribute to PSWs experiencing unplanned absences so that appropriate capacity and supports can be provided to manage the impacts of these unexpected reductions in employees, with ramifications for those who rely on their care provision.

In qualitative and survey-based literature during the COVID-19 pandemic, PSWs highlighted the challenges that they faced when their own unplanned absences resulted in a loss of paid work hours, and how their colleagues’ unplanned absences led to short-staffed teams and increased demands on those who remained (Bandini et al., 2021; Franzosa et al., 2022; Nizzer S. et al., 2022; Pinto et al., 2022). However, beyond sharing the challenges that absenteeism creates, existing literature regarding factors that affect unplanned absences for PSWs is limited; this is particularly true for literature in home care settings and during pandemic scenarios. Two recent reviews have summarized individual and workplace-related factors for the available literature, which mostly focuses on nurses or physicians working in hospital settings pre-pandemic (Brady et al., 2023a, 2023b). Women generally reported more absences than men, with the highest absence rates for women aged 28–37 (Brady et al., 2023b). In terms of workplace characteristics, rates of unplanned absences were higher amongst healthcare workers with lower incomes, living at a further distance from work, and working in facilities with inadequate staffing (Brady et al., 2023a). Mixed results have been found regarding the influence of job tenure on absences for healthcare workers, highlighting a need for further inquiry (Brady et al., 2023a).

Two notable quantitative studies have examined unplanned absences amongst PSWs, generally finding similar factors of influence on absences as reported for other healthcare workers (Gorman et al., 2010; Roland et al., 2022). The first study combined PSWs with other healthcare workers across healthcare sectors to find that female, older, low wage healthcare workers were more likely to take a sickness absence (Gorman et al., 2010). The second found that long-term care home PSWs in the United Kingdom experienced significantly higher rates of unplanned absences with increases in hours worked and distance travelled to work (Roland et al., 2022). Although providing some insight into factors of importance on PSW unplanned absences, these studies used pre-pandemic data, were limited to sickness absence, and did not include findings specific to home care PSWs’ experiences.

Expanding knowledge regarding factors of influence on unplanned absences for the increasingly vital home care PSW occupation is necessary to inform health workforce planning in a context of increasing demands on this sector (Global Coalitation on Aging & Home Instead, 2021). Home care PSW-specific studies are required to account for the differences in worker demographics and workplace factors, compared to other healthcare workers and sectors Zagrodney et al., 2022a, 2022). Understanding these factors would support evidence-based initiatives that account for distinctions between the home care PSW workforce and other healthcare workers and sectors. Targeted data-informed initiatives by government and policymakers aimed at reducing home care PSW unplanned absences can ultimately benefit home care workers and home care recipients.

With the aim of generating insights to inform government and employer policies and practices to reduce unplanned absences for the vital home care PSW workforce, this paper utilized longitudinal administrative quantitative data following PSWs who were employed by a large urban home care organization in Ontario, Canada, to (1) examine the frequency of unplanned absences over time and (2) determine the relative effect of demographic, workplace, and contextual factors on the hazard of taking an unplanned absence in a given week across pre-pandemic and the first three local waves of the pandemic (January 2019–June 2021).

## Methods

The open cohort design utilized weekly longitudinal retrospective administrative data from January 2019 to June 2021, for all PSWs providing publicly funded care within a home care organization delivering care in the Greater Toronto Area (GTA). In addition to describing how unplanned absence rates varied over this period, time-to-event (survival) analysis was used to understand the relative impacts of demographic, workplace, and contextual factors on the odds of experiencing an unplanned absence. Stratified models were used to capture the potential for differing drivers of unplanned absences during this period, which included the onset of the COVID-19 pandemic.

To calculate time-to-event in the analysis, the start date was either on January 1, 2019, for those who were already employed at that time, or on the starting date of employment for those who entered employment after the initial week of the study period within the open cohort. Similarly, the end date (i.e., date of censorship) was either the last week of work at the organization or the last week included within this dataset (June 28, 2021).

Model stratifications were based on definitions of the COVID-19 pandemic waves used by Public Health Ontario (PHO; a provincial-level authority responsible for reporting population-level statistics). The resulting stratifications were pre-pandemic (January 01, 2019–February 26, 2020), early-pandemic (wave one: February 26, 2020–August 31, 2020), and mid-pandemic (waves two and most of three: September 1, 2020–June 30, 2021 (end of sample)) periods (Ontario Agency for Health Protection and Promotion (Public Health Ontario), 2022). Early-pandemic times were considered separately from the subsequent mid-pandemic period to reflect the larger distinction in outcomes, policies, and attitudes between wave one and other waves of the pandemic. The University of Toronto Research Ethics Board reviewed and approved this study (REB#40086).

### Outcome Variable: Unplanned Absences

The outcome variable, unplanned absence, was a binary variable which captured whether a PSW experienced any unplanned absence in a given week. An unplanned absence was defined as unexpectedly missing at least one scheduled client visit (patients are often referred to as clients in home care), whether due to illness, a personal emergency, or other reason. Consecutive weeks with an unplanned absence were treated as separate events. These short-term unplanned absences differed from long-term leaves of absence, which were separate events capturing a PSWs’ planned decision to pause employment and be removed from the work schedule. Although leave of absences are not the focus of this study, descriptive trends in long-term leaves of absences over the study period examined were included to provide contextual information.

### Independent Variables

Individual-level demographic data included PSW age, which was calculated weekly based on birth date, and sex, which was collected in the administrative data as a binary male/female based on employee response at time of hire (gender was not captured). Number of reported spoken languages was included as a control and proxy variable in the absence of other available information capturing ethnicity, racialization, or nationality.

Workplace individual factors included weekly tenure with employer (job tenure) and the number of previous unplanned absences during the study period. Income and travel distance between client homes from the previous week also entered the model to capture how recent work experiences can affect the hazard of an unplanned absence. Income within the past week was divided categorically by third and fourth quartiles versus the reference group of income below the mean. Mean distance travelled per visit within the past week was calculated for each PSW as the total distance that they travelled divided by their total number of visits for that week, which was then categorized by > 1 km (km) versus <1 km.

Geographic work region entered the model to control for jurisdictional differences. Additionally, a variable was constructed to capture geographically defined PSW supply relative to client demand. This ratio was based on the number of clients assigned to a PSW’s care team divided by the number of PSWs within their assigned care team.

Caseload difficulty was controlled for via the inclusion of average age and average health needs of a PSW’s clients in the previous week. Client health needs were classified by their emergency response level ratings, a measure assigned to each client upon entering publicly funded home care, which reflects the number of days that a client can manage safely without formal paid home care support on a scale from 1 to 5 with lower numbers representing more intensive care needs.

Within the stratified models, pandemic-specific contextual factors included weekly COVID-19 case rates reported by PHO (2024) during the pandemic periods and whether public school in-person closures were in effect during a given week due to weather (week of February 12, 2019 (CBC News, 2019)) or pandemic public health policy (March 16, 2020–September 7, 2020; December 21, 2020–February 15, 2021; April 12, 2021–June 14, 2021 (Gallagher-Mackay et al., 2021)).

### Statistical Analysis

**Figure 1.**
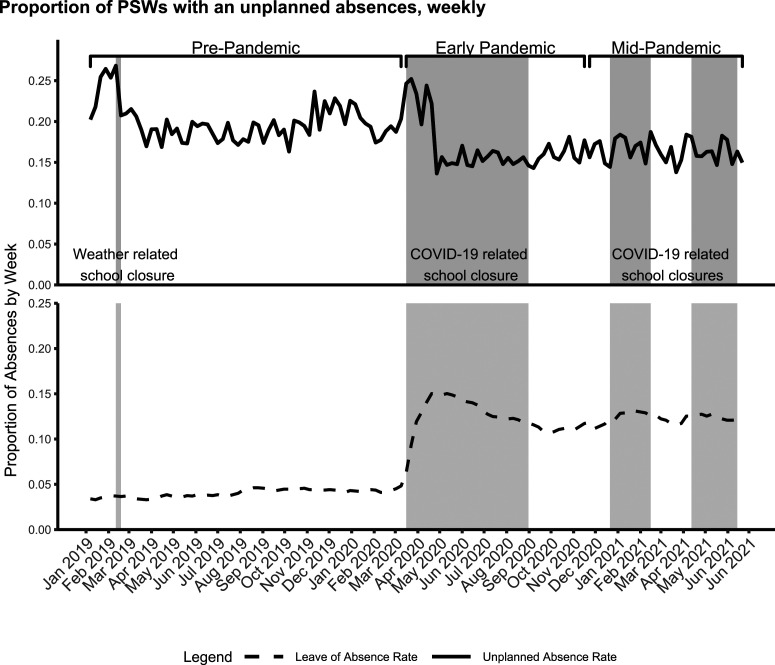
Proportion of PSWs with an unplanned absences, weekly. The solid line representing unplanned absence rate was generated from the number of weekly unplanned absences divided by the number of PSWs currently working within the data. This represents the outcome variable and sample utilized in all analyses. The dotted line representing leave of absence rate was generated from the number of weekly long-term leaves of absence divided by the number of PSWs both currently working and on a leave of absence within the data. This is presented to provide context of larger changes to PSW labor supply in the sample over the time period examined.

Descriptive summaries of the study population consisted of graphical representations (Figure 1), frequency distribution (proportions) or means, and multi- and bivariate tests of significance for differences in proportions of means across all periods (Kruskal–Wallis rank sum test or Pearson’s chi-squared test), early- versus pre-pandemic, mid- versus pre-pandemic (Tables 1 and 2). Bivariate tests of significance (chi-square and t-tests; *p* < .05), stratified by the binomial outcome variable (“Unplanned absence in a given week” vs. “No unplanned absence in a given week”), were also conducted (Table 3).

**Table 1.** PSW Sample Characteristics, Overall and for Each Period.

	Total	Pre-pandemic (Jan 1 2019–Mar 9 2020; 64 weeks)	Early-pandemic (Mar 16 2020–Nov 16 2020; 37 weeks)	Mid-pandemic (Nov 23 2020–Jun 28 2021; 33 weeks)	
	*N* = 1970	*N* = 1663	*N* = 1500	*N* = 1414	
	Mean (SD); *n* (%)	Mean (SD); *n* (%)	Mean (SD); *n* (%)	Mean (SD); *n* (%)	*p-*value^1^
Demographics					
Age	43.85 (10.97)	44.62 (10.79)	46.14 (10.46)***	46.74 (10.44)***	<0.001
Sex					0.8
Female (ref)	1889 (96%)	1604 (97%)	1442 (96%)	1327 (96%)	
Male	72 (4%)	57 (3%)	56 (4%)	55 (4%)	
Number of languages spoken					0.4
English only (ref)	868 (44%)	764 (46%)	663 (44%)	764 (46%)	
English and other languages	1096 (56%)	899 (54%)	837 (56%)	899 (54%)	
Workplace factors					
Client age, mean	62.67 (22.10)	65.93 (18.81)	61.60 (27.28)***	61.07 (28.06)***	0.5
Client needs rating, mean (1 = high needs, 5 = low)	2.81 (0.59)	2.83 (0.60)	2.76 (0.65)**	2.79 (0.61)	0.004
Job tenure (years)	4.70 (5.86)	5.04 (5.96)	5.89 (6.19)***	6.15 (6.34)***	<0.001
Weekly income, categorical					<0.001
Lowest (ref)	1176 (60%)	1019 (61%)	770 (51%)***	663 (47%)***	
Medium	506 (26%)	452 (27%)	378 (25%)***	362 (26%)***	
Highest	282 (14%)	192 (12%)	352 (23%)	389 (28%)	
Weekly distance per visit, continuous	2.40 (2.00)	2.60 (1.95)	2.16 (2.37)	2.13 (2.37)	<0.001
Weekly distance per visit, categorical					<0.001
Low (<1 km/visit)	309 (17%)	168 (11%)	406 (30%)***	382 (27%)***	
High (>1 km/visit)	1561 (83%)	1426 (89%)	944 (70%)***	1062 (73%)***	
Geographic work region					
1 (ref)	813 (41%)	703 (42%)	646 (43%)	579 (43%)	0.2
2	553 (28%)	440 (26%)	421 (28%)	389 (29%)	
3	598 (30%)	520 (31%)	433 (29%)	364 (27%)	
Clients per PSW ratio in local sub-region	2.93 (0.61)	3.14 (0.64)	2.66 (0.58)***	2.83 (0.67)***	<0.001
Outcome variable					
Number of unplanned Absences (days)	23.55 (32.02)	14.35 (17.99)	8.23 (13.22)***	7.21 (11.26)***	<0.001
Average weeks with an unplanned absence (*N*, %)	14.36 (18%)	9.35 (19%)	4.54 (19%)**	4.19 (17%)***	<0.001

^1^Comparison across pre-, early-, and mid-pandemic time periods; Kruskal–Wallis rank sum test or Pearson’s chi-squared test.

*p < .05, **p < .01, or ***p < .001 for test of comparison between pre-pandemic and early-pandemic or pre-pandemic and mid-pandemic (Student’s *t* test, chi-squared; adjusted for multiple comparisons).

**Table 2.** Context Variables by Week, Overall and for Each Period.

	Total (*n* = 131 weeks)	Pre-pandemic (*n* = 64 weeks)	Early-pandemic (*n* = 37 weeks)	Mid-pandemic (*n* = 33 weeks)	*p*-value^1^
Average weekly COVID-19 case rate in Ontario per 100,000 people (mean, SD)	27.94 (46.74)	0.09 (1.01)	22.45 (19.85)	92.56 (54.13)***	<0.001
Weeks with school closures (*N*, %)	56 (43%)	1 (2%)	26 (70%)	19 (58%)**	<0.001

^1^Comparison across pre-, early-, and mid-pandemic time periods; Kruskal–Wallis rank sum test or Pearson’s chi-squared test.

*p < .05, **p < .01, or ***p < .001 for test of comparison between early-pandemic and mid-pandemic (Student’s *t* test, chi-squared; adjusted for multiple comparisons).

**Table 3.** Characteristics of Total PSW Weeks Worked With and Without an Unplanned Absence, Stratified.

	Pre-Pandemic		Early-pandemic		Mid-pandemic	
	Weeks Without and With an Unplanned Absence					
	Without *N* = 66,670	With *N* = 15,554	Without *N* = 36,861	With *N* = 6813	Without *N* = 33,229	With *N* = 5931
	Mean (SD); *n* (%)		Mean (SD); *n* (%)		Mean (SD); *n* (%)	
Demographics^1^						
Age	47.05 (10.34)***	44.52 (10.79)***	47.39 (10.41)***	45.92 (10.90)***	47.76 (10.21)*	46.86 (10.48)*
Sex						
Female	64,301 (97%)	15,058 (97%)	35,344 (96%)	6567 (96%)	31,839 (96%)	5730 (96%)
Male	2278 (3%)	492.00 (3%)	1474 (4%)	243 (4%)	1358 (4%)	200 (4%)
Number of languages spoken						
English only	30,089 (45%)***	8013 (52%)***	16,495 (45%)**	3238 (47%)**	14,263 (43%)***	2769 (47%)***
English and other languages	36,581 (55%)***	7541 (49%)***	20,366 (55%)**	3575 (52%)**	18,966 (57%)***	3162 (53%)***
Workplace factors^1^						
Client age, mean	67.11 (25.92)**	70.79 (21.06)**	61.65 (31.92)***	66.70 (27.21)***	61.30 (32.27)**	70.09 (23.47)**
Client needs rating, mean (1 = high needs, 5 = low)	2.82 (0.66)*	2.86 (0.63)*	2.75 (0.68)	2.76 (0.64)	2.77 (0.65)***	2.84 (0.59)***
Job tenure, years	6.29 (6.31)**	5.46 (5.50)**	6.53 (6.45)***	6.06 (5.95)***	6.59 (6.39)	6.46 (6.05)
Weekly income						
Lowest	33,22 (50%)***	8807 (57%)***	17,071 (46%)***	3519 (52%)***	14,657 (44%)***	2441 (41%)***
Medium	19,984 (30%)***	4490 (29%)***	8756 (24%)**	1656 (24%)**	7426 (22%)***	1663 (29%)***
Highest	13,460 (20%)***	2257 (14%)***	11,034 (30%)***	1,63 (24%)***	11,146 (34%)**	11,034 (30%)**
Distance per visit, continuous	2.56 (2.63)***	2.73 (3.05)***	2.13 (2.84)**	2.31 (3.24)**	2.04 (3.05)**	2.46 (3.75)**
Distance per visit, categorical						
Low (<1 km/visit)	11,265 (18%)***	2402 (16%)***	10,978 (31%)***	1721 (26%)***	11,141 (35%)***	1366 (23%)***
High (>1 km/visit)	51,828 (82%)	12,874 (84%)	24,144 (69%)***	5008 (74%)***	20,429 (65%)***	4460 (76%)***
Geographic work region						
1	29,361 (44%)**	6882 (44%)**	15,889 (43%)**	3067 (45%)**	14,729 (44%)**	2432 (41%)**
2	17,715 (26%)**	3877 (25%)**	10,596 (29%)**	1795 (26%)**	9629 (29%)	1687 (28%)
3	19,594 (29%)**	4795 (31%)**	10,376 (28%)	1951 (29%)	8871 (27%)**	1812 (31%)**
Clients per PSW ratio in local sub-region	3.16 (0.68)**	3.14 (0.65)**	2.65 (0.61)***	2.61 (0.58)***	2.84 (0.69)***	2.80 (0.65)***
Contextual factors^1^						
Average weekly COVID-19 case rate in Ontario per 100,000 people	--	22.47 (19.95)	22.38 (19.35)	92.34 (54.11)	92.34 (54.11)	93.77 (54.21)
School closures	1077 (2%)	256 (2%)	25,137 (68%)*	4746 (70%)*	20,046 (60%)	3626 (61%)

*Note.* Weekly COVID-19 case rates were very low pre-pandemic and were therefore not included in the analysis for the pre-pandemic period.

^1^Factors reflect the week prior. For example, distance travelled in the week prior to a week with or without an unplanned absence.

*p < .05, **p < .01, or ***p < .001 for test of comparison between weeks with and without an unplanned absence within a time period (Student’s *t* test, chi-squared; adjusted for multiple comparisons).

To model recurrent unplanned absences, an extension of a Cox-proportional hazard model was applied. As PSWs could experience multiple absences, which may be statistically correlated with each other (i.e., observations are not independent), a statistical “frailty” term was added to the model to account for individual-level differences not otherwise captured in the data.

To reflect variation in pandemic conditions and public health-related policies during the period studied, three stratified Cox-proportional hazard regression models were used across pre-, early-, and mid-pandemic periods to measure the relative effects of demographic, workplace, and contextual factors on the recurrence of unplanned absences. Model selection was derived from labor economic theory, particularly for absences and women’s labor (Brown & Sessions, 1996; Killingsworth, 1983), which emphasizes the importance of gender differences—particularly in relation to childcare, income, distance travelled at work, the interaction between supply and demand, and external events (e.g., weather and public health conditions). Literature specific to healthcare worker unplanned absence (Brady et al., 2023a, 2023b) also supported the inclusion of such demographic and workplace variables. These factors were included in our models to the extent possible given the scope of available administrative data. An a priori level of statistical significance was set at *p* < .05. All calculations and modelling were performed in *R* version 4.3.0. The hazard ratios and corresponding 95% confidence intervals were estimated. The validity of the proportional hazard assumption was tested graphically, and output is available upon request.

As the large majority of PSWs are female (Zagrodney et al., 2022a), sex differences in sickness absence have been previously reported for healthcare workers (Brady et al., 2023b), and separation of labor economic models by gender is a common practice (Killingsworth, 1983); supplemental models on a female-only cohort were also conducted. Additionally, to test the robustness of our model and sample, sensitivity analyses were conducted by iteratively removing regressors to understand the impacts of correlations between predictors.

## Results

The total number of working PSWs fluctuated throughout the study period with a decline early in the pandemic due to increased leave of absence rates (Figure 1). Amongst the PSWs who remained active in the workforce, there was a sharp spike in unplanned absences during the initial weeks of the pandemic and related school closures (Figure 1). Notably, the spike at the beginning of the pandemic was of similar magnitude to that observed during a winter storm which led to travel challenges and a school closure pre-pandemic (Figure 1).

### Sample Characteristics

Across the full study duration (131 weeks), the open cohort included 1970 unique PSWs each of whom was employed by the organization and provided care during the study period. Individual PSWs were members of the cohort for an average of 83 of the included weeks (range: 1–131). The median age at which a PSW entered the sample (start of study or when hired) was 44 years old. The average age of the cohort was higher during the pandemic (46–47 years) versus the pre-pandemic study period (45 years) (Table 1). The majority of PSWs were female (96%), reflective of the larger home care PSW population in Ontario (34).

#### Influence of Demographics on Unplanned Absences

There was a small but statistically significant protective effect of older age which corresponded to a decreased hazard of taking an unplanned absence. This effect was consistent across all periods (Tables 3 and 4). For every one-year increase in age, there was an approximate 1% decrease in the hazard of taking unplanned absences (HR 0.991; 95% CI [0.987, 0.994]; HR 0.994; 95% CI [0.989, 0.999]; HR 0.992; 95% CI [0.987, 0.998]). There was no statistically significant association between sex and unplanned absences in any period (Tables 2 and 3). Sensitivity analyses which included only female PSWs revealed a similar direction and magnitude of the effect of predictors on unplanned absences. Many PSWs working in our sample could speak another language in addition to English (56%) (Table 1); this was controlled for across all models (Table 3).

**Table 4.** Regression Output for Unplanned Absence, Stratified.

	Pre-pandemic		Early-pandemic		Mid-pandemic	
	HR (95% CI)	*p*-value	HR (95% CI)	*p*-value	HR (95% CI)	*p*-value
Demographics						
Age	0.991 (0.987, 0.994)	<0.001	0.994 (0.989, 0.999)	<0.05	0.992 (0.987, 0.998)	<0.01
Sex						
Female (ref)	1		1		1	
Male	0.867 (0.733, 1.025)	0.056	0.947 (0.723, 1.242)	0.695	0.918 (0.709, 1.189)	0.530
Number of languages spoken						
English only (ref)	1		1		1	
English and other languages	0.869 (0.819, 0.924)	<0.001	0.997 (0.898, 1.107)	0.955	1.004 (0.909, 1.108)	0.939
Workplace factors						
Client age, mean	1.004 (1.003, 1.006)	<0.001	0.999 (0.998, 1.001)	0.418	0.997 (0.995, 0.999)	<0.001
Client needs rating, mean (1 = high needs, 5 = low)	1.040 (0.991, 1.091)	0.110	1.023 (0.945, 1.108)	0.568	1.007 (0.925, 1.096)	0.872
Job tenure (years)	1.000 (0.994, 1.007)	0.940	0.988 (0.978, 0.997)	<0.05	0.988 (0.978, 0.997)	<0.01
Weekly income, categorical						
Lowest (ref)	1		1		1	
Medium	0.945 (0.904, 0.989)	<0.001	0.947 (0.881, 1.019)	0.150	1.031 (0.952, 1.117)	0.453
Highest	0.836 (0.786, 0.889)	<0.001	0.898 (0.824, 0.980)	<0.05	0.879 (0.803, 0.961)	<0.01
Distance per visit, categorical						
Low (<1 km per visit) (ref)	1		1		1	
High (>1 km per visit)	1.166 (1.092, 1.246)	<0.001	1.017 (0.924, 1.119)	0.740	1.252 (1.139, 1.377)	<0.001
Geographic work region						
1 (ref)	1		1		1	
2	0.944 (0.871, 1.025)	0.171	1.028 (0.897, 1.178)	0.650	1.110 (0.976, 1.262)	0.111
3	0.951 (0.872, 1.037)	0.250	1.009 (0.868, 1.171)	0.910	1.13 (0.979, 1.30)	0.095
Clients per PSW ratio in local sub-region	1.006 (0.963, 1.052)	0.770	0.837 (0.771, 0.908)	<0.001	0.928 (0.864, 0.997)	<0.05
Total number of prior weeks with absences	1.084 (1.080, 1.088)	<0.001	1.070 (1.066, 1.074)	<0.001	1.045 (1.042, 1.048)	<0.001
Context variables						
Average weekly COVID-19 case rate in Ontario per 100,000 people	--	--	1.009 (1.004, 1.014)	<0.001	0.996 (0.995, 0.998)	<0.001
School closures						
No (ref)	1		1		1	
Yes	1.597 (1.243, 2.053)	<0.001	0.634 (0.503, 0.799)	<0.001	0.921 (0.790, 1.075)	0.310

#### Influence of Workplace Factors on Unplanned Absences

Factors driven by organizational or governmental policy with a significant effect on unplanned absences were income, distance travelled between clients, caseload characteristics, and client to PSW ratio. PSW income reflects both hours worked and pay rates set by the provincial government in this publicly funded PSW setting. Higher income in the previous week was a significant and substantial predictor of unplanned absences; receiving pay in the highest income bracket corresponded to reductions in the hazard of taking an unplanned absence by 10.2%–16.4% (pre: HR 0.836; 95% CI [0.786, 0.889]; early: HR 0.898; 95% CI [0.824, 0.980]; mid: HR 0.879; 95% CI [0.803, 0.961]). PSWs who travelled further between clients (averaging >1 km vs. <1 km) saw an increase in the hazard of taking an unplanned absence in the following week in both pre- (16.6%) and mid- (25.2%) pandemic periods (HR 1.166; 95% CI [1.092, 1.246]; HR 1.252; 95% CI [1.139, 1.377], respectively). Home care PSWs caring for incrementally older clients had a small but significantly higher hazard of unplanned absences during pre-pandemic times (HR 1.004; 95% CI [1.003, 1.006]) and a small but significantly lower hazard of unplanned absences mid-pandemic (HR 0.997; 95% CI [0.995, 0.999]). Although there were significantly higher unplanned absences for PSWs caring for clients with higher care needs when considered independently (Table 2), there was no significant effect once accounting for other factors in the regression (Table 3). Client age and client care needs were not highly correlated and sensitivity analyses with care needs removed did not change the significance nor direction of client age in the model. Higher localized client to PSW ratios correlated with a decrease in the hazard of an unplanned absence by 7.2%–16.3% during pandemic periods (HR 0.837; 95% CI [0.771, 0.908]; HR 0.928; 95% CI [0.864, 0.997]).

While not directly modifiable by the organization, the number of previous weeks with unplanned absences and job tenure were additional work-related factors which had a significant impact on PSWs’ unplanned absences. An increase in the number of previous weeks with an unplanned absence corresponded to a 4.5%–8.5% increase in the hazard of taking an unplanned absence, with higher hazard ratios pre-pandemic (HR 1.084; 95% CI [1.080, 1.088]), compared to early- (HR 1.070; 95% CI [1.066, 1.074]) or mid- (HR 1.045; 95% CI [1.042, 1.048]) pandemic. For each additional year, job tenure was associated with a 1% decrease in the hazard of taking an unplanned absence during early- and mid-pandemic periods (Table 3) (HR 0.988; 95% CI [0.978, 0.997]; HR 0.988; 95% CI [0.978, 0.997], respectively). The effect of job tenure remained fairly stable in sensitivity analyses testing for potential multicollinearity when age was removed (HR 1.000 vs. HR 0.999; HR 0.988 vs. HR 0.982; HR 0.988 vs. HR 0.980).

#### Influence of Contextual Factors

Provincial COVID-19 case rates had statistically significant but minimal impacts on PSWs’ unplanned absences in early- (0.9%) and mid- (−0.4%) pandemic models (HR 1.009; 95% CI [1.004, 1.014]; HR 0.996; 95% CI [0.995, 0.998]). The single school closure due to inclement weather that occurred pre-pandemic corresponded with a large (59.7%) and significant effect on the hazard of a PSW taking an unplanned absence (HR 1.597; 95% CI [1.243, 2.053]). Prolonged pandemic-related school closures correlated with a reduction in unplanned absences early-pandemic (HR 0.634; 95% CI [0.503, 0.799]).

## Discussion

The stratified models reflect factors impacting unplanned absences during pre-, early-, and mid-pandemic periods using longitudinal administrative PSW data from a large urban home care organization. Factors most strongly correlating with lower rates of unplanned absences across multiple periods were higher income, shorter travel distance between clients, and increased age. Based on these findings, unplanned absences for home care PSWs could be expected to decrease if policies and practices are implemented which increase home care PSW incomes and reduce travel distances between clients. Implications of any resultant improvements to stability of home care PSWs include benefits to society (Gianino et al., 2019), employers (Kocakulach et al., 2016), PSWs themselves (Brady et al., 2023a), and their clients—particularly given the known benefits of continuity of care (Reckrey et al., 2024; Russell et al., 2011).

The proportion of PSWs with an unplanned absence spiked early in the pandemic before dropping below the pre-pandemic baseline, coinciding with a large number of PSWs initiating prolonged leaves of absence (Figure 1). This pattern reflects similar trends for other healthcare workers internationally during this time (e.g., in the United Kingdom (Edge et al., 2021)). The reduced short-term unplanned absence rate for most of the pandemic also matches historical patterns of a decline in healthcare worker absences during periods of economic instability and high unemployment (Brady et al., 2023a) (as was the case in Ontario at this time (Clarke & Fields, 2022)). Despite potential for increased exposure to COVID-19, relatively high adherence to facial protective equipment reported by home care PSWs during this time (King et al., 2022) may have also contributed to reduced illness-related unplanned absences.

Home care PSWs in the present study experienced more unplanned absences than PSW samples who reported absences due to illness or injury across sectors (hospital, long-term care home, and home care sectors combined) (23.6 days in our sample vs. 19.6 days (Blackwell, 2023)). Differences in the average number of PSW absences could be due to variations in how absences are defined across sectors; our home care data counts even a single missed care visit for any reason (sickness or otherwise) as an absence in a given day versus other sources which may define an absence as missing a full 12-hour scheduled shift (a more common schedule pattern in other sectors) and reported absences limited to illness reasons. The difference in absence rates may also be reflective of the different working environments between institutional and non-institutional settings, including shift versus fee-for-service payment models, co-location of clients which removes the need for travel, and higher wages in institutional settings (Zagrodney et al., 2023a, 2023b). The higher rate of unplanned absences for home care PSWs suggests a need for targeted policies and practices to reduce unplanned absences in order to limit consequences for PSWs, care team members, employers, and those relying on their care.

Although COVID-19 case rates were a significant predictor of unplanned absences, the effect was relatively small. In addition to reflecting the prevalence of COVID-19 infection within the community, it is possible that some of the effects captured within this time-dependent variable were reflective of other events, behaviors, and attitudes which changed over time. Interestingly, when the COVID-19 case rate variable was removed from the model during sensitivity analyses, the effect of school closure policies became more prominent. Patterns of unplanned absences in this study as they relate to age and school closures are likely reflective of the generally higher level of unpaid caregiving responsibilities provided by home care PSWs (Zagrodney et al., 2022a). Although PSWs were not explicitly asked in available data whether school closures were the reason for their unplanned absences, the timing of each major school closure had strong correlations with unplanned absences. The timing of school closures was distinct from other contextual factors, including COVID-19 case rates, where previous research (Boothe et al., 2022) and our supplementary analysis found no strong correlation between Ontario school closures and COVID-19 case rates. Prior to the pandemic, the single school closure during the study period was weather-related and corresponded with a 60% increased hazard of an unplanned absence, which may have reflected a combination of PSWs’ difficulty in travelling to clients and lack of childcare. During the pandemic, the jurisdiction examined (Ontario, Canada) experienced one of the highest number of weeks with school closures, internationally, and the highest in Canada (Gallagher-Mackay et al., 2021). The reduced hazard of experiencing an unplanned absence correlated with the prolonged school closures during pandemic periods of the study period may indicate self-selection of PSWs with childcare responsibilities who took leaves of absence following the onset of the pandemic (Figure 1). Indeed, data on leaves of absence for home care PSWs during the pandemic have shown that childcare responsibilities were a primary driver of new leaves of absences during this period, increasing from a pre-pandemic baseline of 3% to a high of 18% of the home care PSW workforce at this organization (Figure 1) (King et al., 2023). Additionally, school closures corresponded with other closures such as community senior centers offering adult day services; this could have unintended effects on increased caregiving pressures for PSWs who were family caregivers to older adults as well (Wister & Kadowaki, 2021). The effect of school closures found in this study provides novel evidence for government policymakers, allowing them to better understand consequences of school closures on this highly gendered workforce to other parts of the economy like the home care sector.

Across all periods, younger PSWs were also more likely to take unplanned absences, consistent with previous findings in nursing populations (Brady et al., 2023b). The higher average age of PSWs in early- and mid-pandemic periods versus pre-pandemic (Table 1) also indicates that younger PSWs, who are generally more likely to have childcare responsibilities, temporarily or permanently exited the workforce during this time. A major contributing factor to this and similar results of higher unplanned absences for predominantly female occupations (96% in this sample) may be the “double burden” gender divide in family responsibilities with a high reliance on younger women (<50) to also provide unpaid family caregiving tasks (Bratbergy et al., 2002). Consistent with this gendered caregiver responsibility interpretation wherein unplanned absences are reoccurring in correspondence with changing childcare responsibilities, PSWs in this study with a greater number of previous unplanned absences were 5–8% more likely to experience further unplanned absences; research on other healthcare workers has found similar effects (Brady et al., 2023a). These findings, coupled with prior research, highlight how PSWs participate in both paid and unpaid aspects of the care economy, and the potential impact that changes to one portion of the care economy (e.g., childcare through school closures) can have on other elements of the care economy (i.e., health care) (Morgan et al., 2022). PSW employers may consider offering childcare services when schools are closed or benefit programs specific to unpaid caregivers to help reduce the impacts of potential for double burden.

From the perspective of workforce stability, employers also have an opportunity to manage distance travelled between clients as a way of reducing unplanned absences. Travel between client homes represents a substantial part of home care PSWs’ work experience and is frequently noted as a challenge in the qualitative literature (Muramatsu et al., 2018; Nizzer et al., 2022). Travelling more than 1 km per visit, on average, had a significant and large (16.6%–25.2%) effect on unplanned absences the following week during pre- and mid-pandemic periods. In our sample, pre-pandemic travel between clients averaged 2.6 km, dropping to 2.2 km during the pandemic, partially due to the implementation of operational initiatives to reduce travel (McKay et al., 2023) and the non-significance of travel distance early-pandemic may indicate different decision-making priorities during this unique period.

Consistent with labor supply models on incentivization to work (Brown & Sessions, 1996), receiving the highest level of income was protective against the hazard of an unplanned absence. Additionally, that the number of PSWs in the highest income quartile more than doubled following the onset of the pandemic (Table 1) may be attributable to government policies for PSW-specific wage enhancements (Government of Ontario, 2020), employment limitations requiring healthcare workers to choose a single employer rather than continuing to hold multiple part-time or casual jobs (as is common amongst PSWs, particularly in home care (Zagrodney et al., 2022b)), in addition to employer-based operational efficiencies which reduced travel time and distance during the pandemic allowing for more paid hours of work (McKay et al., 2023). These findings highlight how organizational and government policy decisions impacting income can not only have potential effects on (Ontario Community Support Association OCSA, 2022) long-term supply, but also short-term supply through unplanned absences.

The pandemic resulted in changes to both who was receiving and who was delivering care (King et al., 2023), where this new balance shifted toward fewer clients per PSW, on average, during the pandemic compared to the pre-pandemic period (Table 1). During the pandemic, PSWs who continued to provide care and had higher client to PSW ratios were less likely to have an unplanned absence. This finding is consistent with qualitative studies in which PSWs reported feelings of responsibility to provide care to vulnerable individuals during the pandemic (Nizzer et al., 2022).

### Limitations

The administrative data utilized in this study was originally collected for the purposes of service planning and delivery at a single publicly funded home care organization in an urban region in Ontario, Canada. Common limitations with secondary use of administrative data sets apply, with limited availability of additional explanatory contextual variables. For instance, reasons for unplanned absences were not clearly recorded. Reasons recorded administratively may reflect strategic reporting of absence types based on differing levels of compensation (e.g., the first two days of personal emergency leave are fully paid, sickness absences are compensated as short-term disability rather than at a full daily rate, and a generic “unplanned absence” is not compensated). Some additional factors that may influence PSW absences—including ethnicity, race, and nationality—were unavailable in the data. During the study period, many new public health policies and changing experiences of, and attitudes toward, COVID-19 occurred. While it is not possible to capture the distinct effects of each of these policies independently within a single model, COVID-19 case rates and school closures were selected based on prior qualitative research as most likely to reflect PSWs’ experiences during this time (Nizzer et al., 2023). Further, model stratification allowed us to capture differences in factors influencing rates of PSW unplanned absences between pre-, early-, and mid-pandemic periods. Model stratification reduces potential effects of selection bias due to changes in workforce composition across the study period, as the effect of each independent variable is examined for PSWs working within a more limited time frame.

## Conclusions

Findings presented in this paper provide insight for evidence-informed interventions related to the influence of socio-demographic, workplace, and contextual factors on home care PSW unplanned absences in pandemic and non-pandemic periods. This is an increasingly critical topic in health workforce planning that otherwise has not received much attention. Based on these results, policies aimed at increasing income and reducing travel distance between clients are expected to improve home care PSW labor supply via reductions to unplanned absences. This highly gendered workforce, whose members are more likely to have additional unpaid family caregiving responsibilities, was strongly impacted by school closures. This finding highlights the impact of disruptions in the broader care economy on this vital component of the healthcare workforce. As demand for home care continues to grow, improving stability of home care access and PSW supply through reducing unplanned absences will be increasingly beneficial for PSW themselves and for those relying on PSW care to remain safe at home.

## References

[bibr1-07334648251316973] Bandini J. Rollison J. Feistel K. Whitaker L. Bialas A. Etchegaray J. (2021). Home care aide safety concerns and job challenges during the COVID-19 pandemic. New Solutions: A Journal of Environmental and Occupational Health Policy, 31(1), 20–29. 10.1177/1048291120987845 33451266

[bibr2-07334648251316973] Blackwell A. J. (2023). Quality of employment and labour market dynamics of health care workers during the COVID-19 pandemic. Statistics Canada. Retrieved from: https://www150.statcan.gc.ca/n1/pub/75-006-x/2023001/article/00007-eng.htm

[bibr3-07334648251316973] Boothe K. Fiorillo N. Just D. Alvarez E. Davidson A. (2022). School closure decisions in Alberta and Ontario during COVID-19: Discourse and data. Canadian Journal of Political Science/Revue canadienne de science politique, 55(3), 740–746. 10.1017/S000842392200049X

[bibr4-07334648251316973] Brady H. D. McGrath D. Dunne C. P. (2023a). Sick leave determinants in the healthcare sector (Part I): A review of contextual factors. Brown Hospital Medicine, 2(1), 2. 10.56305/001c.57688 PMC11878852 40046536

[bibr5-07334648251316973] Brady H. D. McGrath D. Dunne C. P. (2023b). Sick leave determinants in the healthcare sector (Part III): A review of individual-level factors. Journal of Brown Hospital Medicine, 2(3), 2. 10.56305/001c.77844 PMC11864490 40026473

[bibr6-07334648251316973] Bratberg E. Dahl S. Å. Risa A. E. (2002). The double burden’: Do combinations of career and family obligations increase sickness absence among women? European Sociological Review, 18(2), 233–249. 10.1093/esr/18.2.233

[bibr7-07334648251316973] Brown S. Sessions J. G. (1996). The economics of absence: Theory and evidence. Journal of Economic Surveys, 10(1), 23–53. 10.1111/j.1467-6419.1996.tb00002.x

[bibr8-07334648251316973] Canadian Institute for Health Information (CIHI) . (2023). Hospital staffing and hospital harm trends throughout the pandemic. CIHI. Retrieved from: https://www.cihi.ca/en/hospital-staffing-and-hospital-harm-trends-throughout-the-pandemic#ref6

[bibr9-07334648251316973] CBC News . (2019). Massive winter storm hitting eastern Ontario. CBC News.

[bibr10-07334648251316973] Clarke S. Fields A. (2022). Employment growth in Canada and the United States during the recovery from COVID-19. Statistics Canada. Retrieved from: 10.25318/36280001202201200001-eng

[bibr11-07334648251316973] Deloitte . (2021). Canada’s elder care crisis: Addressing the doubling demand. In Canadian medical association (CMA). Retrieved from: https://www.cma.ca/sites/default/files/pdf/health-advocacy/activity/CMA-LTC-Deloitte-Report-EN.pdf (Accessed 13 Apr 2022).

[bibr12-07334648251316973] Edge R. van der Plaat D. A. Parsons V. Coggon D. van Tongeren M. Muiry R. Madan I. Cullinan P. (2021). Changing patterns of sickness absence among healthcare workers in England during the COVID-19 pandemic. Journal of Public Health, 44(1), e42–e50. 10.1093/pubmed/fdab341 PMC8499865 34514506

[bibr13-07334648251316973] Franzosa E. Wyte-Lake T. Tsui E. K. Reckrey J. M. Sterling M. R. (2022). Essential but excluded: Building disaster preparedness capacity for home health care workers and home care agencies. Journal of the American Medical Directors Association, 23(12), 1990–1996. 10.1016/j.jamda.2022.09.012 36343702 PMC9634621

[bibr14-07334648251316973] Gallagher-Mackay K. Srivastava P. Underwood K. Dhuey E. McCready L. Born K. Maltsev A. Perkhun A. Steiner R. Barrett K. (2021). COVID-19 and education disruption in Ontario: Emerging evidence on impacts.

[bibr15-07334648251316973] Gianino M. M. Politano G. Scarmozzino A. Stillo M. Amprino V. Di Carlo S. Benso A. Zotti C. M. (2019). Cost of sickness absenteeism during seasonal influenza outbreaks of medium intensity among health care workers. International Journal of Environmental Research and Public Health, 16(5), 747. 10.3390/ijerph16050747 30832264 PMC6427598

[bibr16-07334648251316973] Global Coalition on Aging . (2021). Building the caregiving workforce: Our aging world needs. Global Coalition on Aging. Retrieved from: https://globalcoalitiononaging.com/wp-content/uploads/2021/06/GCOA_HI_Building-the-Caregiving-Workforce-Our-Aging-World-Needs_REPORT-FINAL_July-2021.pdf

[bibr17-07334648251316973] Gorman E. Yu S. Alamgir H. (2010). When healthcare workers get sick: Exploring sickness absenteeism in British Columbia, Canada. Work, 35(2), 117–123. 10.3233/WOR-2010-0963 20164606

[bibr18-07334648251316973] Government of British Columbia . (2020). Pandemic pay supports front-line health, social service workers. Ministry of Finance: Victoria. Retrieved from: https://news.gov.bc.ca/releases/2020FIN0031-000907

[bibr19-07334648251316973] Government of Ontario . (2020). Ontario provides $461 million to temporarily enhance wages for personal support workers. Queen's Printer for Ontario, pp. 2012–2021. Retrieved from: https://news.ontario.ca/en/release/58627/ontario-provides-461-million-to-temporarily-enhance-wages-for-personal-support-workers

[bibr20-07334648251316973] Government of Saskatchewan . (2020). Temporary wage supplement for lower income essential workers for vulnerable citizens. Government of Saskatchewan.

[bibr21-07334648251316973] Killingsworth M. R. (1983). Labor supply (1). Cambridge university press Cambridge.

[bibr22-07334648251316973] King E. C. Zagrodney K. A. P. McKay S. M. Holness D. L. Nichol K. A. (2022). Determinants of nurse's and personal support worker's adherence to facial protective equipment in a community setting during the COVID-19 pandemic in Ontario, Canada: A pilot study. American Journal of Infection Control. 10.1016/j.ajic.2022.07.021 PMC9338445 35917934

[bibr23-07334648251316973] King E. C. Zagrodney K. A. P. Rabeenthira P. Van Belle T. A. McKay S. M. (2023). Why did home care personal support service volumes drop during the COVID-19 pandemic? The contributions of client choice and personal support worker availability. Health Services Insights, 16(1), 11786329231210692. 10.1177/11786329231210692 38028120 PMC10644723

[bibr24-07334648251316973] Kocakulah M. C. Kelley A. G. Mitchell K. M. Ruggieri M. P. (2016). Absenteeism problems and costs: Causes, effects and cures. International Business & Economics Research Journal, 15(3), 89–96. 10.19030/iber.v15i3.9673

[bibr25-07334648251316973] McKay S. Konan M. Tedesco S. Turrif T. Michener M. King E. C. (2023). Optimizing weekend schedules in home health care: The essential care on weekends for personal support quality improvement project. Home Health Care Management & Practice, 1(1), 10848223231183091. 10.1177/10848223231183091

[bibr26-07334648251316973] Morgan R. Tan H.-L. Oveisi N. Memmott C. Korzuchowski A. Hawkins K. Smith J. (2022). Women healthcare workers’ experiences during COVID-19 and other crises: A scoping review. International Journal of Nursing Studies Advances, 4(1), 100066. 10.1016/j.ijnsa.2022.100066 35128472 PMC8801061

[bibr27-07334648251316973] Muramatsu N. Sokas R. K. Chakraborty A. Zanoni J. P. Lipscomb J. (2018). Slips, trips, and falls among home care aides: A mixed-methods study. Journal of Occupational and Environmental Medicine, 60(9), 796. 10.1097/JOM.0000000000001355 29787398 PMC6125748

[bibr28-07334648251316973] Nizzer S. Ruco A. Moreira N. King E. McKay S. Nichol K. Holness D. L. (2022). Homecare personal support worker experiences working during the COVID-19 pandemic: A qualitative study. Safety and Health at Work, 13(1), S176–S177. 10.1016/j.shaw.2021.12.1303

[bibr29-07334648251316973] Nizzer S. Ruco A. Moreira N. A. Holness D. L. Nichol K. A. King E. C. McKay S. M. (2023). “You have to be careful about every detail” How the COVID-19 pandemic shaped the experiences of Canadian personal support workers working in home care. Journal of Occupational and Environmental Medicine, 65(9), e604–e609. 10.1097/JOM.0000000000002911 37365749 PMC10487389

[bibr30-07334648251316973] Ontario Agency for Health Protection and Promotion (Public Health Ontario) . (2022).Weekly epidemiologic summary: COVID-19 in Ontario – september 18, 2022 to september 24, 2022. King’s Printer for Ontario.

[bibr31-07334648251316973] Ontario Community Support Association (OCSA) . (2022). 2022 pre-budget consultation submission. In 7 Reasons to Care: Why we need to invest in Ontario's home and community care sector to safeguard the future sustainability of our health system. Retrieved from: https://www.ocsa.on.ca/2022-ontario-pre-budget-submission

[bibr32-07334648251316973] Pinto A. D. Hapsari A. P. Ho J. Meaney C. Avery L. Hassen N. Jetha A. Lay A. M. Rotondi M. Zuberi D. , (2022). Precarious work among personal support workers in the greater Toronto Area: A respondent-driven sampling study. Canadian Medical Association Open Access Journal*,* 10(2), E527-E538. 10.9778/cmajo.20210338 PMC9343122 35700996

[bibr33-07334648251316973] Public Health Ontario (PHO) . (2022). Ontario respiratory virus tool. Government of Ontario. Retrieved from: https://www.publichealthontario.ca/en/Data-and-Analysis/Infectious-Disease/Respiratory-Virus-Tool

[bibr34-07334648251316973] Reckrey J. M. Russell D. Fong M.-C. Burgdorf J. G. Franzosa E. C. Travers J. L. Ornstein K. A. (2024). Home care worker continuity in home-based long-term care: Associated factors and relationships with client health and well-being. Innovation in Aging, 8(3), igae024. 10.1093/geroni/igae024 38505005 PMC10946305

[bibr35-07334648251316973] Roland D. Allan S. Chambers E. Smith D. Gousia K. , (2022). Personal assistants in England and the factors associated with absenteeism. Frontiers in Public Health. 10.3389/fpubh.2022.970370 PMC9589044 36299742

[bibr36-07334648251316973] Russell D. Rosati R. J. . Rosenfeld P. Marren J. M. . (2011). Continuity in home health care: Is consistency in nursing personnel associated with better patient outcomes? Journal for Healthcare Quality, 33(6), 33–39. 10.1111/j.1945-1474.2011.00131.x 22103703

[bibr37-07334648251316973] Sinn C.-L. J. Sultan H. Turcotte L. A. McArthu C. Hirdes J. P. (2022). Patterns of home care assessment and service provision before and during the COVID-19 pandemic in Ontario, Canada. PLoS One, 17(3), Article e0266160. 10.1371/journal.pone.0266160 35353856 PMC8966998

[bibr38-07334648251316973] Wister A. Kadowaki L. (2021). Social isolation among older adults during the pandemic. Employment and Social Development Canada. In Her majesty the queen in right of Canada, 2021. Cat. No.: Em12-82/2021E-PDF ISBN: 978-0-660-41256-6.

[bibr39-07334648251316973] Zagrodney K. A. P. King E. C. Simon D. Nichol K. A. McKay S. M. (2023a). A good investment: Expanding capacity to care for older adults in the home and community care sector through increased personal support worker wages. Canadian Journal on Aging/La revue canadienne du vieillissement, 2(4), 1–6. 10.1017/S0714980823000557 37721030

[bibr40-07334648251316973] Zagrodney K. A. P. King E. C. Simon D. Nichol K. A. McKay S. M. (2023b). Economic evidence for home and community care investment: The case for Ontario personal support workers' wage parity. Healthcare Policy, 19(1), 23–31. 10.12927/hcpol.2023.27161 37695703 PMC10519337

[bibr41-07334648251316973] Zagrodney K. A. P. Deber R. Saks M. Laporte A. (2022a). Personal support worker socio-demographic differences across care sectors in Canada. Journal of Applied Gerontology, 42(4), 670–679. 10.1177/07334648221142301 36464973 PMC9996787

[bibr42-07334648251316973] Zagrodney K. P. A. Deber R. Saks M. Laporte A. , (2022b). The disadvantaged home care personal support worker: Differences in job characteristics, unionization, pensions, participation, and wages by care sector in Canada. Journal of Applied Gerontology, 42(4), 758–767. 10.1177/07334648221146260 36524373 PMC9996786

